# Medical News and Miscellany

**Published:** 1896-02

**Authors:** 


### Medical News and Miscellany.

---

**Dr. E. E. Johnson** has removed from Denison to Honey Grove, Texas.

---

**Dr. A. P. Griffin,** of Whitney, Texas, was married to Miss Stella Middlebrooks on January 10th.

---

"**Enclosed** find P. O. money order for two dollars ('sound money') for renewal of subscription for the irresistible 'Red Back.' Yours truly, JAS. M. FRY."

---

**Advertisement.** — The TEXAS MEDICAL JOURNAL, issued monthly at Austin, has the highest circulation rating accorded to any periodical of its class in the State.—*From Printers' Ink, issue of May 8, 1895.*

---

**Dr. J. O. Williams,** late of Wm. Penn, Texas, has just returned from a course at the Philadelphia Polyclinic, and has located in Houston; being succeeded at Wm. Penn by Dr. E. M. Albers, late of Victoria.

**The** JOURNAL acknowledges the receipt of cards announcing the marriage, February 5th inst., at Cameron, Texas, of Miss Mabel May Lott, daughter of Dr. M. K. Lott, to Mr. Felix C. Humphries, of Belton, Texas.

---

**Dr. H. H. Darr,** of Caldwell, has been quite ill for several months, and laid up for repairs at Fort Worth, under the care of Dr. F. D. Thompson. We are pleaseed to learn he has been repaired and is now nearly as good as new.

---

**Dr. Z. T. Bundy,** of Milford, Texas, is taking a special course at the New York Polyclinic. He writes: "Seeing the familiar Red Back in the reading-room of the Polyclinic I felt like I had met an old friend and did not feel lonesome. Send it to me here for the present."

---

**Dr. T. D. Wooten,** of Austin, has had erected a splendid building, and he and his two sons, Drs. G. H. and J. S. Wooten, have had it furnished and equipped with modern appliances for the treatment of surgical and gynecological cases. The infirmary is first class in every respect.

---

**The** "Red Back" has a warm place in the hearts of its readers: Dr. Abney, of Franklin, remitting for advance subscription says: "I don't consider my office complete without the 'Red Back.' May it live long and prosper in its well directed mission in promoting the science of medicine."

---

**The Students' Council Journal.**—*The University Medical* shows marked improvement thus early in its career; the last issue being very sprightly indeed. The JOURNAL desires to express its congratulations to "the boys" and the hope that the *Medical* may continue to grow and improve. The Red Back will have to look to its laurels.

---

**Erratum.**—In our last issue it was stated that Dr. W. F. Starley, Jr., of Tyler, had been appointed resident student at the Sealy hospital. That was a mistake; Dr. Starley was elected by the Faculty of the Texas Medical College to the responsible position of Resident Physician and Superintendent,—quite a compliment and worthily bestowed.

---

**"Blocks of Three."**—To induce prompt renewals and new subscriptions, the JOURNAL makes this offer for 1896: The subscription price is $2.00 a year. We will send three copies one year for $4.00, all new subscribers, or one renewal and two new names. That is, any one, subscriber no not, who will send us two new subscribers and $4.00, will receive the JOURNAL one year *free* of charge.

---

GALVESTON, TEXAS, Dec. 21, 1895.

*Editors Texas Medical Journal:*

Please mention for me in the next issue of the JOURNAL that in consequence of the inferior paper used in wrapping, several copies of the transactions have been returned to me with the address torn off. If any member has failed to receive his book, it will give me pleasure to promptly forward it to him upon notification of the fact.

H. A. WEST, Sec'y T. S. M. A.

---

**Dr. Geo. Dock,** for some years Professor of Medicine in the University of Michigan, has been appointed Professor of Pathology and Bacteriology in Jefferson Medical College, Philadelphia. Dr. Dock is a graduate of the Jefferson, and when fresh from college, came to Texas to take a professorship in the Texas Medical College, being recommended by the Jefferson Faculty as one of their brightest students. To attain to so high a position in his famous *alma mater*, and so young, too, is a most distinguished honor. We congratulate the doctor.

---

**We regret** to see from the last issue of the *Southwest Texas Medical Reporter*, that friend Bell has taken exceptions to our notice of that publication in our Jannary number. We disclaim all thought or intention of reflecting on the *Reporter* or its esteemed editors. Surely, the doctor did not take to himself the *N. Y. Medical Record's* editorial—which we reproduced—on "Bogus Medical Journals." We do not put the *Hygiea* or its successor in that category; and Dr. Bell must see that what the *Record* says is *true*, of those publications that have no *raison d'etré*. The *Reporter* has.

---

**Dr. S. E. Milliken,** of New York, published a report of an operation, "tendon grafting for infantile paralysis," and sent out reprints of same, embellished with his portrait and biography. Now comes Dr. B. F. Parrish, in the *New York Medical Record*, and claims priority for the operation, and asserts that Milliken knew of the case and his report of same,—a pretty strong arraignment. In defense of his claim, Dr. Milliken says, in the *N. Y. Medical Record*, January 25th, that Parrish slit up the tendon, while he slit it down; Parrish cut it an inch or more, he cut it only an inch; Parrish used catgut sutures, and he used kangaroo sutures; so, you see? And, then, Parrish didn't *embellish* his (a strong point of dissimilarity which the doctor didn't mention)! Just published it "plain so"; no trimmin's.

---

The JOURNAL owes and hereby tenders an apology to its esteemed correspondent, Dr. W. M. Yandell, for the unpardonable manner in which the statistics in his paper in last issue was mutilated by want of proper attention on the part of the proof reader, and we call attention to the following errors:

ERRATA.—In Dr. W. M. Yandell's paper on "Diphtheria and Sewer Gas," published in our last issue,—January—in the table at foot of page 356—make the following corrections: For "Nationality: Mexicans 53, Americans 2, Negroes 2; total 25," read "Deaths: Mexicans 14, Americans 9, Negroes 2; total 25. Nationality: Mexicans 53, Americans 34, Negroes 6; total 93." On page 357, paragraph 3, for "complete," read "completely." On page 358, last word in 4th line from top, for "excepted," read "exempt."

---

**Heavy Death Rate.**—Dr. T. J. Turpin, State Quarantine Inspector at Laredo, writes that according to the report to the Supervising Surgeon General M. H. S., there were, during the year ending December 31, 1895, 331 deaths in Laredo, of whom 314 were Mexicans, 16 Americans, and 1 colored (American). Infants under 3 years of age, 148. There were 113 (about one-third) died without having had medical attention. The diseases were: Chronic diarrhœa, 22; "convulsion" (cholera infantum), 25; meningitis, 11; typhoid fever, 4; "fever," 8; cholera, infantum, 19; whooping cough, 8; consumption, 14; enteritis, 5; marasmus, 6; pneumonia, 21; remittent fever, 1; lockjaw, 2; infantile lockjaw, 14; still born (?), 11; croup, 1; rheumatism, 2; heart disease, 12; puerperal fever, 4; dysentery, 4; syphilis, 2; cancer, 4; appendicitis, 1; jaundice (?), 1.

Estimated population, 11,000. Ratio per 1000 per annum, 30.9.

The doctor says the city physician is only paid $25 a month salary.

Such a state of affairs, the doctor says, is disgraceful to any community calling itself civilized. He is not the city physician and has nothing to do with local sanitation. He advises that relief committees should be organized to hunt out the sick and destitute and give them relief; the churches, for instance, should take it in hand. The city physician should be paid a salary which would enable him to give his time and attention to such matters. The people should be enlightened by lectures on hygiene, etc. It is a sad state of affairs, but hardly worse, we suppose, than other towns with so large a Mexican population. The lower classes of Mexicans are recklessly indifferent to dirt, disease and death. Dr. Turpin thinks that fully one-half of these deaths might have been prevented by proper means.

---

**The Congress of Medico-Climatology,** to be held at San Antonio, Texas, this month, February 20–23, has been well advertised, and it is expected that there will be a large attendance. Interest in climate as related to health, is receiving great and growing attention. Climate is being studied with a view to practical results,—not only as a matter of comfort and pleasure to those in health, but as an element of prophylaxis, where there is predisposition to disease, either hereditary or acquired, and of therapeutics, an element in the cure of those affected with disease. The time will come, and soon, when a doctor can prescribe "climate" with the same degree of confidence and precision that he now prescribes any other remedy,—or, preventive. The climate of San Antonio has long been famous as antagonistic to consumption, and there are thousands living to-day, whose life has been prolonged and health restored by a residence there; thousands perhaps reclaimed from the clutches of the fell destroyer. San Antonio is, therefore, an ideal place in which to meet and discuss climate.

Dr. J. A. Robinson, of Chicago, is president of the Congress. There are five vice-presidents, and all but one from Chicago, and four Chicago secretaries, two "recording" and two "corresponding," and the treasurer is from Chicago also. Chicago physicians are taking a big interest in this meeting. (They will be wanting to move San Antonio, or, at least, its climate, there, next step, or make a "model" of it.)

The local physicians of San Antonio have organized into a committee of arrangement and reception, and will lay themseves out to entertain delegates. We trust there may be a large attendance. A trip to San Antonio is worth making for itself alone, but with such an attraction a visit will be made doubly interesting. Nearly all the State medical societies have appointed delegates. For information relative to the organization, address Dr. A. F. McKay, Chicago,—one of the secretaries.

